# Book Notices

**Published:** 1868-09-01

**Authors:** 


### *BOOK NOTICES.*

A Theoretical and Practical Treatise on Midwifery, including the Diseases of Pregnancy and Parturition. By P. Cazeaux, Member of the Imperial Academy of Medicine, etc., etc., Paris. Revised and annotated by S. Tarnier, Adjunct Professor in the Faculty of Medicine, Hospital Surgeon, etc., etc., Paris. Fifth American, from the seventh French edition, by Wm. R. Bullock, M.D. One hundred and seventy Illustrations. Philadelphia: Lindsay & Blakiston. 1868. Pp. xxxi. 1124. Chicago: S. C. Griggs & Co. Cloth, $6.50; leather, $7.50.

There are few books on this subject more widely known or more highly appreciated than this. It is unnecessary to enlarge upon the merits of the original editions by the celebrated author, whose comparatively recent demise the whole professional world laments. It is or should be in the library of every student and practitioner. The present edition has undergone at the hands of M. Tarnier many and important alterations and additions. Cazeaux's text is in large type—Tarnier's portion being indicated by a smaller letter. A single glance at the book will show that the latter's office has been no sinecure. Aside from additions, the plan of the whole has been so greatly modified so as to make it essentially a new work. Full credit is awarded to contemporaries, especially where new theories or procedures are proposed.

On the whole, this may be well considered as the most complete and advanced record of Obstetrical science and Gynæcology that is in the possession of the profession.

It is needless to say that the eminent American publishers have done their work excellently well. We trust they may be rewarded by the abundant sale this production so preëminently deserves.

---

THE ANATOMY AND HISTOLOGY OF THE HUMAN EYE. By A. METZ, M.D., Professor of Opthalmology in Charity Hospital Medical College, Cleveland, Ohio. Philadelphia: Published at the Office of the *Philadelphia Medical and Surgical Reporter*. 8vo. pp. xvi, 184. Price, $2.50, postage paid.

This is an exhaustive treatise on the subject, and yet both compact and clear. The older and more voluminous treatises on the Anatomy and Histology of the Eye, whilst wearisome to read, do not furnish a tithe of the matter this work contains. From the array of authorities (about forty) credited in the primary pages, we are promised the very latest and best information, and this promise perusal of the book shows to be religiously fulfilled. There are seventy-five neat and accurate illustrations, with lucid descriptions. The whole is issued in creditable style by the publishers. We congratulate the author on the successful accomplishment of the effort announced in his preface, and the publisher on its elegant "make up."

---

KLINIK DER OHRENKRANKHEITEN. Ein Handbuch für Studirende und Aerzte, von Dr. S. Moos, Praktischer Arzt und docent an der Universität ein Heidelberg. Mit 26 in den Text Gedruckten Holzschnitten; Wien, 1866, Wilhelm Braumüller.

Those of our readers who are familiar with the German language, will find this an excellent work of reference in the case of Diseases of the Ear.

---

PROGRESSIVE LOCOMOTOR ATAXIA. Being extracts from two papers on this topic, read before the Wood County Medical Association, Parkersburg, W. Virginia. By WALTER COLES, M.D., Professor of Diseases of Women and Children in Medical College of Virginia.

The author in this tractate brings together the prominent characteristics of this much to be dreaded disease, and brings its pathological history up to the present time.

---

CHICAGO: Past, Present and Future. By JOHN S. WRIGHT. Chicago: Western News Co. 8vo. pp. 404.

Those interested (and who is not?) in this Metropolis of the North-west, will find herein recorded the reasons why its growth has taken place, and why it must continue. The author writes *con amore*, and with the confidence of one who can truthfully say *quorum pars fui*, having been one of the earliest settlers. It is illustrated by maps, views of public buildings, etc., etc. The task of compiling the array of facts and figures given must have been a very onerous one, and we have every reason to believe has been executed with perfect integrity and fidelity. Unbelievers in the continued prosperity and stability of this city, have only carefully to read and ponder this book to become convinced that Chicago will, at no distant day, rank as the second city on the continent. Buy it and read.

